# A Phase Ib, open-label, dose-finding study of alpelisib in combination with paclitaxel in patients with advanced solid tumors

**DOI:** 10.18632/oncotarget.25854

**Published:** 2018-08-03

**Authors:** Jordi Rodon, Giuseppe Curigliano, Jean-Pierre Delord, Wael Harb, Analia Azaro, Yu Han, Celine Wilke, Valerie Donnet, Dalila Sellami, Thaddeus Beck

**Affiliations:** ^1^ Molecular Therapeutics Research Unit, Department of Medical Oncology, Vall d’Hebron University Hospital, Centro Cellex, 08035, Barcelona, Spain; ^2^ Division of Early Drug Development for Innovative Therapies, Department of Hematology and Oncology, University of Milano, Istituto Europeo di Oncologia, 20141, Milan, Italy; ^3^ Clinical Research Unit, Institut Claudius Regaud, 31052, Toulouse, France; ^4^ Horizon Oncology Center, 47905, Lafayette, IN, USA; ^5^ Novartis Pharmaceuticals Corporation, 07936, East Hanover, NJ, USA; ^6^ Novartis Pharma AG, Postfach, CH-4002, Basel, Switzerland; ^7^ Novartis Pharma S.A.S., 92506, Rueil-Malmaison, France; ^8^ Highlands Oncology Group, 72703, Fayetteville, AR, USA

**Keywords:** breast neoplasms, drug resistance, *PIK3CA* protein, human, chemotherapy

## Abstract

Phosphatidylinositol 3-kinase (PI3K) pathway activation is associated with resistance to paclitaxel in solid tumors. We assessed the safety and activity of alpelisib, an oral, selective PI3K p110α inhibitor, plus paclitaxel in patients with advanced solid tumors. This Phase Ib, multicenter, open-label, dose-finding study, with a planned dose-expansion phase of alpelisib once daily (QD) plus fixed-dose paclitaxel, recruited patients with advanced solid tumors. For the dose-finding phase, the primary objective was determination of maximum tolerated and/or recommended Phase II dose of alpelisib plus paclitaxel, and the secondary objectives included the assessment of safety for this combination. From March 2014 to August 2016, 19 patients with advanced solid tumors were treated with alpelisib QD (300 mg, n=6; 250 mg, n=4; 150 mg, n=9) plus paclitaxel (80 mg/m^2^, per standard of care). During dose finding, five of 12 (41.7%) evaluable patients for MTD determination experienced dose-limiting toxicities: alpelisib 300 mg, Grade 2 hyperglycemia (n=1); alpelisib 250 mg, Grade 2 hyperglycemia (n=1), Grade 4 hyperglycemia and Grade 3 acute kidney injury (n=1); and alpelisib 150 mg, Grade 2 hyperglycemia (n=1) and Grade 4 leukopenia (n=1). The MTD of alpelisib when administered with paclitaxel was 150 mg QD. Most frequent all-grade AEs were diarrhea (73.7%; Grade 3/4 10.5%) and hyperglycemia (57.9%; Grade 3/4 31.6%). The planned dose-expansion phase was not initiated. Alpelisib plus paclitaxel has a challenging safety profile in patients with advanced solid tumors. This study was closed following the completion of the dose-finding phase. Clinical trial registration: ClinicalTrials.gov NCT02051751.

## INTRODUCTION

Although paclitaxel, a taxane-based chemotherapy, has been approved as a weekly injection for the treatment of several solid tumors [1], most patients develop resistance and progressive disease [2, 3]. Resistance to paclitaxel has been associated with phosphatidylinositol 3-kinase (PI3K)/mechanistic target of rapamycin (mTOR) pathway activation *in vitro* and *in vivo* [4–6]. However, the administration of a PI3K inhibitor has increased sensitivity in paclitaxel-resistant prostate cancer cells [6]. In addition, decreased tumor burden was observed in mice inoculated with ovarian cancer cells and treated with a PI3K inhibitor plus paclitaxel compared with those treated with paclitaxel alone [5].

Activation of the PI3K/mTOR pathway frequently occurs via mutations in *PIK3CA*, which encodes the class I PI3K p110α catalytic subunit [7]. Further, *PIK3CA* mutations are among the most frequently observed alterations in solid tumors [8–12]. Therefore, administration of paclitaxel plus a PI3K p110α-specific inhibitor is a potential therapeutic strategy by which to delay disease progression in patients with advanced solid tumors.

Alpelisib (BYL719) is an oral, selective inhibitor of class I PI3K p110α that has shown potent antitumor activity in preclinical studies [13, 14]. In a Phase Ia study of single-agent alpelisib in patients with advanced solid tumors (NCT01219699), a tolerable safety profile and encouraging preliminary activity was observed in those with *PIK3CA*-altered (mutation/amplification) advanced solid tumors, with the maximum tolerated dose (MTD) declared as 400 mg once daily (QD) [15, 16]. In addition, single-agent alpelisib was generally well tolerated, with the most frequent all-grade, treatment-related adverse events (AEs; ≥30% of patients) including hyperglycemia (51.5%), nausea (50.0%), decreased appetite (41.8%), diarrhea (40.3%), and vomiting (31.3%) [15, 16]. Hyperglycemia is an expected on-target side effect of PI3Kα inhibition given the involvement of PI3Kα in glucose homeostasis regulation and occurs more frequently with a targeted compound like alpelisib compared to pan-PI3K inhibitors or to beta, gamma or delta specific PI3K inhibitors. The most frequent all-grade AEs (≥30% of patients) reported with a 1-hour intravenous (IV) infusion of paclitaxel 80 mg/m^2^ once weekly (QW) included fatigue (47%), alopecia (36%), abdominal pain (33%), nausea (31%), and peripheral neuropathy (31%) [17].

Here, we report the results from a Phase Ib dose-finding study, which evaluated the MTD of alpelisib QD when administered with paclitaxel in patients with advanced solid tumors, and discuss the safety profile of this combination.

## RESULTS

### Patient characteristics and disposition

From March 5, 2014, to August 19, 2016, 19 patients with advanced solid tumors were treated with alpelisib QD plus paclitaxel during dose finding, as follows: alpelisib 300 mg (n=6); alpelisib 250 mg (n=4) and alpelisib 150 mg (n=9). Median age was 57 years and the most common primary sites of cancer were breast (26%) and rectum (16%). The number of metastatic sites was one or two in most patients (Table 1).

**Table 1:** Patient demographics and disease characteristics at baseline

	All patients N=19
**Median age, years (range)**	57.0 (27.0–76.0)
**Male, n (%)**	7 (36.8)
**Race, n (%)**	
Caucasian	19 (100)
**ECOG performance status, n (%)**	
0	10 (52.6)
1	9 (47.4)
**Primary site of cancer, n (%)**	
Breast	5 (26.3)
Cartilage	1 (5.3)
Cervix	1 (5.3)
Malignant thymoma	1 (5.3)
Ovary	1 (5.3)
Pancreas	1 (5.3)
Prostate	1 (5.3)
Rectum	3 (15.8)
Small cell lung cancer	1 (5.3)
Soft tissue	1 (5.3)
Stomach	1 (5.3)
Unknown origin	2 (10.5)
**Number of metastatic sites, n (%)**	
1	6 (31.6)
2	8 (42.1)
3	3 (15.8)
≥4	2 (10.5)

ECOG, Eastern Cooperative Oncology Group.

At data cut-off (August 19, 2016), all patients had discontinued study treatment, primarily due to disease progression (63.2%) (Table 2). Three patients (15.8%) experienced AEs leading to discontinuation of one or both study drugs, as follows: one discontinued both drugs due to Grade 3 dehydration, acute kidney injury, and hyperglycemia (alpelisib 250 mg cohort); one discontinued both drugs due to Grade 4 neutropenia and γ-glutamyltransferase increase (alpelisib 150 mg cohort); and one discontinued paclitaxel only due to Grade 2 peripheral neuropathy (alpelisib 150 mg cohort).

**Table 2:** Patient disposition

	All patients N=19
**Treatment ongoing, n (%)**	0
**Treatment discontinued, n (%)**	19 (100.0)
**Primary reason for treatment discontinuation, n (%)**	
Disease progression	12 (63.2)
Patient decision	3 (15.8)
Adverse events	2 (10.5)
Physician decision	2 (10.5)

Data cut-off: August 19, 2016.

### Dose finding and dose-limiting toxicities

Of the 12 patients evaluable for MTD determination, five (41.7%) experienced DLTs (Table 3): one patient in the alpelisib 300 mg cohort; two in the alpelisib 250 mg cohort, and two in the alpelisib 150 mg cohort. DLTs included leukopenia, hyperglycemia, and acute kidney injury. Analysis supported an MTD of alpelisib 150 mg QD when administered with paclitaxel 80 mg/m^2^ QW, which had the highest posterior probability of being within the target toxicity interval (16%, 35%) while also meeting the EWOC criterion.

**Table 3:** Dose-limiting toxicities in patients treated with alpelisib 150–300 mg QD plus paclitaxel 80 mg/m^2^ QW

	Alpelisib 150 mg QD + paclitaxel	Alpelisib 250 mg QD + paclitaxel	Alpelisib 300 mg QD + paclitaxel	All patients
DLTs, n (%)	n=8	n=3	n=1	N=12^a^
**Total**	2 (25.0)	2 (66.7)	1 (100)	5 (41.7)
Leukopenia	1 (12.5)	0	0	1 (8.3)
Hyperglycemia	1 (12.5)	2 (66.7)^b^	1 (100)	4 (33.3)^b^
Acute kidney injury	0	1 (33.3)^b^	0	1 (8.3)^b^

DLTs, dose-limiting toxicities; QD, once daily; QW, once weekly.

^a^ Seven patients were not evaluable due to not experiencing a DLT in Cycle 1 and inadequate drug exposure.

^b^ Grade 4 hyperglycemia and Grade 3 acute kidney injury DLTs were experienced by the same patient.

### Safety and tolerability

The median duration of exposure (range) to study treatment for each dose level was as follows: alpelisib 300 mg QD, 8.9 weeks (4.0–16.1); alpelisib 250 mg QD, 18.9 weeks (1.0–76.6); and alpelisib 150 mg QD, 24.1 weeks (8.0–80.1). The most frequent all-grade treatment-emergent AEs (≥40% of patients) regardless of relationship were diarrhea (n=14 [73.7%]), hyperglycemia (n=11 [57.9%]), anemia (n=8 [42.1%]), asthenia (n=8 [42.1%]), and nausea (n=8 [42.1%]) (Table 4). The most frequent Grade 3/4 treatment-emergent AEs (≥10% of patients) regardless of relationship were hyperglycemia (n=6 [31.6%]), anemia (n=2 [10.5%]), diarrhea (n=2 [10.5%]), lymphopenia (n=2 [10.5%]), neutropenia (n=2 [10.5%]), and leukopenia (n=2 [10.5%]) (Table 4).

**Table 4:** Treatment-emergent adverse events (≥20% of all patients, all grades) regardless of relationship to study drug

Adverse events, n (%)	Alpelisib 150 mg + paclitaxel (n=9)		Alpelisib 250 mg + paclitaxel (n=4)		Alpelisib 300 mg + paclitaxel (n=6)		All patients (N=19)	
	Grade ≥3	All-grade	Grade ≥3	All-grade	Grade ≥3	All-grade	Grade ≥3	All-grade
**Total**	3 (33.3)	9 (100.0)	3 (75.0)	4 (100.0)	5 (83.3)	6 (100)	11 (57.9)	19 (100)
Diarrhea	1 (11.1)	6 (66.7)	0	3 (75.0)	1 (16.7)	5 (83.3)	2 (10.5)	14 (73.7)
Hyperglycemia	1 (11.1)	2 (22.2)	3 (75.0)	4 (100)	2 (33.3)	5 (83.3)	6 (31.6)	11 (57.9)
Anemia	1 (11.1)	6 (66.7)	0	1 (25.0)	1 (16.7)	1 (16.7)	2 (10.5)	8 (42.1)
Asthenia	0	5 (55.6)	0	1 (25.0)	0	2 (33.3)	0	8 (42.1)
Nausea	0	5 (55.6)	0	1 (25.0)	0	2 (33.3)	0	8 (42.1)
Fatigue	0	5 (55.6)	0	0	0	2 (33.3)	0	7 (36.8)
Lymphopenia	1 (11.1)	3 (33.3)	1 (25.0)	2 (50.0)	0	2 (33.3)	2 (10.5)	7 (36.8)
Neutropenia	1 (11.1)	4 (44.4)	0	1 (25.0)	1 (16.7)	2 (33.3)	2 (10.5)	7 (36.8)
Alopecia	0	5 (55.6)	0	0	0	1 (16.7)	0	6 (31.6)
Decreased appetite	0	1 (11.1)	0	2 (25.0)	0	3 (50.0)	0	6 (31.6)
Leukopenia	1 (11.1)	3 (33.3)	0	1 (25.0)	1 (16.7)	2 (33.3)	2 (10.5)	6 (31.6)
Weight decreased	0	1 (11.1)	0	1 (25.0)	0	4 (66.7)	0	6 (31.6)
Peripheral neuropathy	0	4 (44.4)	0	1 (25.0)	0	0	0	5 (26.3)
Peripheral edema	0	4 (44.4)	0	0	0	1 (16.7)	0	5 (26.3)
Stomatitis	0	2 (22.2)	0	2 (50.0)	0	1 (16.7)	0	5 (26.3)
Vomiting	0	2 (22.2)	0	2 (50.0)	0	1 (16.7)	0	5 (26.3)
Hypokalemia	1 (11.1)	1 (11.1)	0	2 (50.0)	0	1 (16.7)	1 (5.3)	4 (21.1)
Rash	0	3 (33.3)	0	1 (25.0)	0	0	0	4 (21.1)

Data cut-off: August 19, 2016.

The most common reason for both alpelisib dose reduction and interruption was AEs. The rate of alpelisib dose reduction (at least one) was highest in patients treated with alpelisib 300 mg (66.7%), followed by those with alpelisib 250 mg (50.0%) and alpelisib 150 mg (22.2%). The rate of alpelisib dose interruption was highest in patients treated at the 250 mg dose level (75.0%), followed by those at both the 300 mg (66.7%) and 150 mg (66.7%) dose levels. AEs leading to study drug discontinuation were reported in three patients (15.8%). One patient discontinued paclitaxel due to Grade 2 neuropathy, but continued to receive alpelisib, one patient discontinued study treatment due to Grade 4 neutropenia and Grade 4 gamma-glutamyltransferase increase, and one patient discontinued study treatment due to Grade 3 dehydration, Grade 3 acute kidney injury, and Grade 2 and Grade 3 hyperglycemia.

There was one on-treatment death (during treatment or within 30 days of last study treatment dose): a patient died due to progression of small cell lung cancer on Day 101, 18 days after the last administration of alpelisib.

### Pharmacokinetics

**Figure 1:**
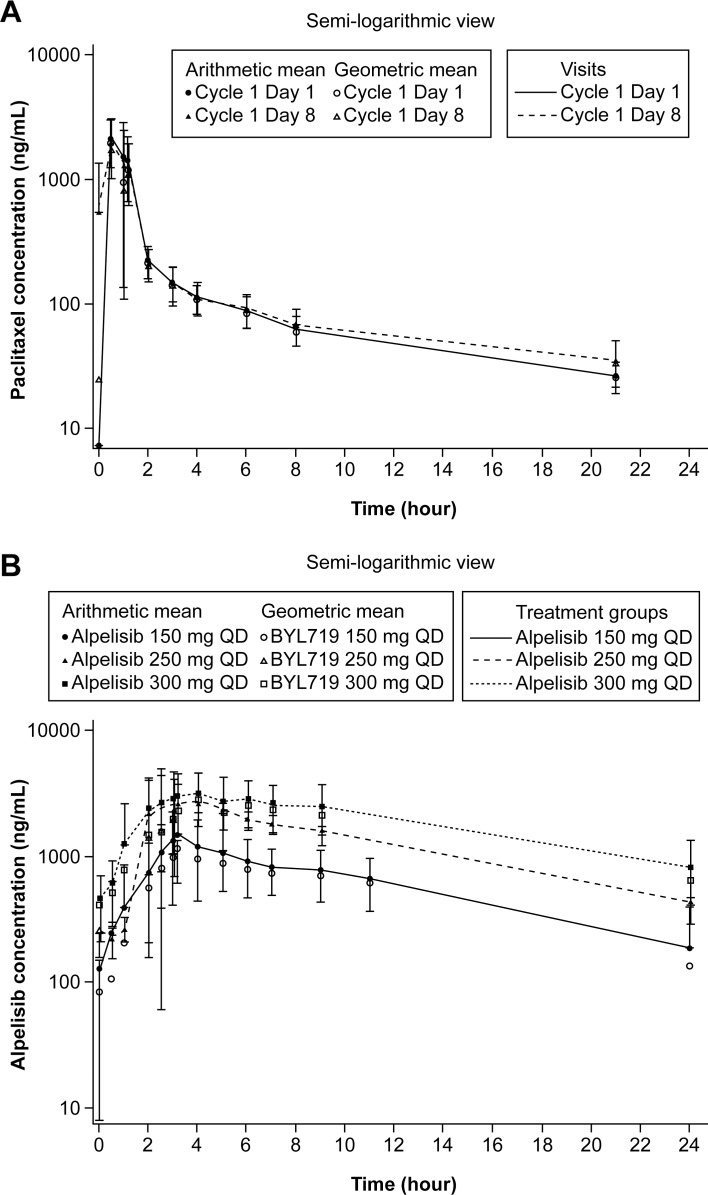
Geometric mean and arithmetic mean (SD) concentration-time profiles (pharmacokinetic analysis set) **(A)** paclitaxel by visit and **(B)** plasma alpelisib by dose level at Cycle 1 Day 8. QD, every day. Data cut-off: August 19, 2016.

Similar concentration–time profiles of paclitaxel were observed in the presence and absence of alpelisib and independent of alpelisib dose (Figure 1). The plasma drug exposure (area under the curve from time zero to infinity [AUC_inf_] and maximal drug concentration [C_max_]) of paclitaxel were comparable on Cycle 1 Day 1 and Cycle 1 Day 8, and were independent of dose level, except for C_max_ for alpelisib 300 mg on Cycle 1 Day 8, which was lower and more variable (Table 5). Median time to maximum concentration (T_max_) of alpelisib on Cycle 1 Day 8 ranged from 3.17 to 4.17 hours, independent of alpelisib dose (Table 5). C_max_ and AUC from time zero to 24 hours (AUC_0-24_) after the oral administration of alpelisib increased in a dose-dependent manner.

**Table 5:** Primary pharmacokinetic parameters for paclitaxel at Cycle 1 Day 1 and Cycle 1 Day 8, and alpelisib at Cycle 1 Day 8^a^

	Alpelisib 150 mg + paclitaxel		Alpelisib 250 mg + paclitaxel		Alpelisib 300 mg + paclitaxel	
**Paclitaxel**						
	C1D1	C1D8	C1D1	C1D8	C1D1	C1D8
AUC_inf_, geometric mean ng·hr/ml (CV%) [n]	4360 (26.7) [7]	4370 (22.8) [8]	4320 (43.4) [3]	5000 (42.7) [2]	5000 (24) [5]	4470 (37.8) [4]
C_max_, geometric mean ng/ml (CV%) [n]	2650 (32.8) [8]	2790 (26.3) [9]	2770 (56.4) [3]	2960 (16.1) [3]	2700 (19.5) [5]	1250 (114) [6]
T_max_, median hours (range) [n]	1 (0.87–1.17) [8]	1 (0.5–1.07) [9]	1 (0.45–1.05) [3]	0.8 (0.5–1.17) [3]	1 (0.5–1.28) [5]	0.75 (0–2.67) [6]
**Alpelisib**						
	C1D8		C1D8		C1D8	
AUC_0–24_, geometric mean ng·hr/ml (CV%) [n]	13800 (36.8) [9]		27700 (7.78) [2]		37100 (66.4) [5]	
C_max_, geometric mean ng/ml (CV%) [n]	1390 (63.7) [9]		3750 (51.6) [2]		3000 (63.8) [6]	
T_max_, median hours (range) [n]	3.17 (1–8.92) [9]		3.23 (2.47–4) [2]		4.17 (3–6) [6]	

AUC_0–24_, area-under the curve from time 0 to 24 hours; AUC_inf_, area-under the curve from time 0 to infinity; C1D1, Cycle 1 Day 1; C1D8, Cycle 1 Day 8; C_max_, maximal drug concentration; CV, geometric coefficient of variation; T_max_, time to maximal drug concentration.

^a^ Pharmacokinetic analysis set.

Data cut-off: August 19, 2016.

### Clinical activity

**Figure 2:**
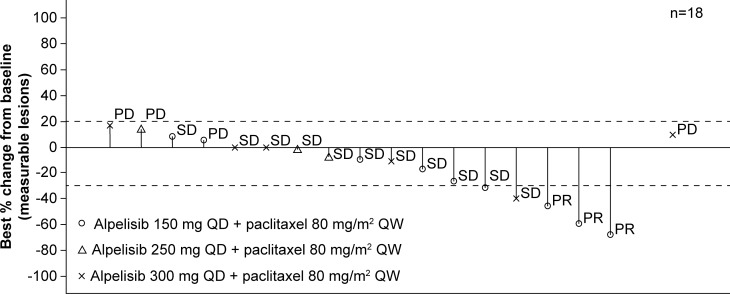
Waterfall plot of tumor responses Best percentage change from baseline in sum of longest diameters and best overall response per local investigator assessment. PD, progressive disease; PR, partial response; QD, every day; QW, every week; SD, stable disease. ^*^One patient who did not have any target lesion at baseline has been excluded from the graph. Missing bar denotes a missing percentage change from baseline. Data cut-off: August 19, 2016.

Best overall response and best percentage change from baseline per local investigator assessment were evaluated in 18 patients with measurable lesions (Figure 2). Best overall responses were partial response in three patients, stable disease in 11 patients and progressive disease in four patients.

## DISCUSSION

The MTD of alpelisib was 150 mg QD when administered with paclitaxel 80 mg/m^2^ QW in patients with solid tumors; however, the overall tolerability of this regimen was challenging. In contrast, the MTD of single-agent alpelisib in a first-in-human study in patients with solid tumors was higher at 400 mg QD, with a manageable safety profile [15, 16].

Hyperglycemia was the most frequent DLT during dose finding, occurring in four of 12 evaluable patients (33.3%). In all patients treated with alpelisib 150–300 mg QD plus paclitaxel (N=19), hyperglycemia was the most frequent Grade 3/4 AE. The occurrence of hyperglycemia in the present study was consistent with that in patients treated with single-agent alpelisib, where hyperglycemia was also the most frequent DLT during dose finding, occurring in seven of 68 evaluable patients (10.3%) [16]. In patients treated with single-agent alpelisib 30–450 mg QD or 120–200 mg twice daily (N=134), hyperglycemia was also the most frequent all-Grade AE suspected to be treatment related (69 [51.5%]) and Grade 3/4 AE (32 [23.9%]) [16]. Hyperglycemia is an expected on-target side effect of PI3Kα inhibition given the involvement of PI3Kα in glucose homeostasis regulation [13–19]. In the present study, hyperglycemia was managed with oral antidiabetic medications (e.g. metformin; insulin considered for higher grades) and dose interruption of alpelisib followed by re-starting the same dose or undergoing dose reduction depending on the initial grade of hyperglycemia and subsequent fasting plasma glucose level. Other AEs were managed by concomitant medications and alpelisib dose interruption and/or reduction. The rates of dose reduction and interruption were high across all dose levels, although these rates should be interpreted with caution given the small cohort sizes. It should also be noted that the administration of corticosteroids as a premedication was not standardized, which prevented their contribution to the rates of AEs, namely hyperglycemia, from being determined. AEs leading to study drug discontinuation were reported in three patients; in two of the patients these AEs included ones previously reported for weekly paclitaxel (peripheral neuropathy and neutropenia) at the same dose in this study [20].

Plasma drug exposure to alpelisib increased in a dose-dependent manner and was generally comparable to the steady-state exposure of single-agent alpelisib at similar dose levels [16]. The plasma drug exposure of paclitaxel was generally comparable both on Cycle 1 Day 1 and Cycle 1 Day 8, and was independent of alpelisib dose, showing that steady-state exposure of alpelisib 150–300 mg had no impact on paclitaxel metabolism.

Preliminary efficacy results for alpelisib 150 mg QD plus paclitaxel are inconclusive; in a Phase I study, single-agent alpelisib exhibited preliminary anti-tumor activity in solid tumors from doses of 270 mg QD and higher [15, 16]. Therefore, given the challenging safety profile of alpelisib plus paclitaxel, the potentially limited clinical activity of alpelisib at 150 mg QD [15, 16], and the emergence of new treatment options for patients with breast cancer, the planned dose-expansion phase of this study was not initiated and the intermittent administration of alpelisib not investigated. The present study was therefore closed following the completion of the dose-finding phase.

In contrast to this study, the combination of alpelisib with nab-paclitaxel was well tolerated in a Phase I study (N=10) [21]. The MTD of alpelisib was not reached and the recommended Phase II dose was alpelisib 350 mg daily plus nab-paclitaxel 100 mg/m^2^ IV weekly [21]. Although the results need to be verified in a larger cohort, this study indicates that adding alpelisib to a taxane may be possible.

Finally, promising disease control and survival benefits, with a manageable safety profile, have been observed with alpelisib plus fulvestrant in a Phase Ib study (NCT01219699) in patients with *PIK3CA*-altered estrogen receptor-positive, HER2–advanced breast cancer (ABC) [22, 23]. Accordingly, the Phase III SOLAR-1 study (NCT02437318) is investigating the efficacy and safety of alpelisib 300 mg QD plus fulvestrant in patients with *PIK3CA*-mutant and wild-type, aromatase inhibitor-resistant, hormone receptor-positive (HR+), HER2–ABC. Further, the Phase II BYLieve study (NCT03056755) is investigating the efficacy and safety of alpelisib 300 mg QD plus fulvestrant or letrozole in patients with *PIK3CA* mutant, HR+, HER2–ABC, who have progressed on or after cyclin-dependent kinase 4/6 inhibitor treatment. The challenging safety profile of alpelisib plus paclitaxel from the present closed study will help inform ongoing studies of alpelisib in combination with other therapies.

## MATERIALS AND METHODS

### Study design

This Phase Ib, multicenter, open-label, dose-finding study explored escalating doses of alpelisib plus fixed-dose paclitaxel in patients with advanced and unresectable solid tumors. A dose-expansion phase was planned in patients with human epidermal growth factor receptor 2-negative (HER2–) locally advanced or metastatic breast cancer, or with recurrent or metastatic head and neck squamous cell carcinoma (HNSCC) resistant to platinum-based chemotherapy.

In the dose-finding phase, the primary objective was to determine the MTD and/or recommended Phase II dose (RP2D) of alpelisib QD when administered with paclitaxel 80 mg/m^2^ QW in patients with advanced solid tumors, and the secondary objectives were to assess safety and tolerability and to characterize the pharmacokinetic (PK) profile.

### Patient population

Key inclusion criteria for the dose-finding part of the study included age ≥18 years; at least one measurable or non-measurable lesion per Response Evaluation Criteria In Solid Tumors (RECIST) v1.1; tumor tissue available for PI3K signaling analysis; adequate bone marrow and organ function; histologically confirmed, advanced unresectable solid tumors and progression on (or unable to tolerate) standard therapy within 3 months before screening, or no available standard anticancer therapy; and Eastern Cooperative Oncology Group (ECOG) performance status ≤2. Prior antineoplastic therapy (including taxanes) was permitted provided the patient recovered from related side effects to ≤Grade 1. Key exclusion criteria included previous treatment with a PI3K or AKT inhibitor (mTOR inhibitors allowed); peripheral sensory neuropathy with functional impairment (Grade ≥2); impaired cardiac function or significant cardiac disease; concurrent treatment with medication with a known risk of QT prolongation or inducing torsades de pointes; diabetes mellitus requiring insulin treatment and/or with clinical signs; impaired gastrointestinal (GI) function or GI disease that may significantly alter alpelisib absorption; or human immunodeficiency virus, active hepatitis B and/or C infection.

All patients provided written informed consent. The study was conducted in accordance with the Declaration of Helsinki and guidelines for Good Clinical Practice, as defined by the International Conference on Harmonization.

### Protocol amendments

The study was initiated on March 5, 2014. A first protocol amendment (June 23, 2014; after six patients had been treated) allowed two additional starting doses of alpelisib; other amendments included changing the permitted fasting plasma glucose value at screening from ≤140 mg/dL to ≤120 mg/dL, modifying the dose-limiting toxicity (DLT) grading of hyperglycemia for consistency with Common Terminology Criteria for AEs (CTCAE) v4.03, and updating the management of some AEs to align with the overall clinical program. The main purpose of the second amendment (January 28, 2015; after 14 patients had been treated) was to modify the guidelines for pneumonitis management.

### Treatment

Patients received oral alpelisib QD in 28-day cycles (300, 250, or 150 mg) and paclitaxel 80 mg/m^2^ QW as a 1-hour (±15 minutes) IV infusion after standard premedication (per local standard practice). Patients received treatment until disease progression, unacceptable toxicity, patient decision, death, or discontinuation for any other reason.

### Assessments

Safety was monitored by physical examination and assessment of vital signs, weight, and performance status; and by performing electrocardiogram, cardiac imaging, ophthalmic, and laboratory evaluations. AEs were assessed continuously and graded per CTCAE v4.03. Radiologic response was evaluated by computed tomography or magnetic resonance imaging by the local investigator per RECIST v1.1 at baseline and 8-weekly intervals. PK samples for alpelisib were collected from all patients. PK samples for paclitaxel were taken on Cycle 1 Day 1/2 before, during, and after the end of infusion, and again on Cycle 1 Day 8/9 after seven doses of alpelisib. Treatment with alpelisib was initiated on Cycle 1 Day 2, and PK samples were taken during steady state on Cycle 1 Day 8/9 before, during and after the influence of premedication and paclitaxel to assess impact on alpelisib exposure under conditions with the highest risk of an interaction.

### Analysis sets

Analysis sets were as follows: full analysis set (FAS), all patients who received at least one dose of alpelisib and/or paclitaxel; safety set, all patients who received at least one dose of alpelisib and/or paclitaxel and had at least one valid post-baseline safety assessment; dose-determining set (DDS), all patients from the safety set who during Cycle 1 either met the minimum exposure criterion and had sufficient safety evaluations, or experienced a DLT; and pharmacokinetic analysis set, all patients in the FAS who received at least one dose of alpelisib and/or paclitaxel and had at least one evaluable concentration measurement.

### Statistical analyses

An adaptive 5-parameter Bayesian logistic regression model (BLRM) with escalation with overdose control (EWOC) guided the dose-finding of each combination [18]. The EWOC principle only recommends doses for which the risk of overdosing (true DLT rate >0.35) is <25% for the next dose level. DLTs were defined as AEs or abnormal laboratory values considered to be unrelated to disease, disease progression, inter-current illness, or concomitant medications that occurred within the first cycle of treatment and met any of the criteria listed in Supplementary Table 1. AEs that did not meet the criteria within Supplementary Table 1 but led to a dose interruption of alpelisib for >7 consecutive days, or paclitaxel for ≥2 consecutive doses within Cycle 1, or between Cycle 1 and Cycle 2 Day 1, were also defined as DLTs. Available data were analyzed after Cycle 1 per cohort of patients to decide the dose for the next cohort and/or determine the MTD/RP2D. MTD estimation was based on the dose-dependent incidence rate of DLTs in Cycle 1 for patients of the DDS using the BLRM with EWOC principle.

## SUPPLEMENTARY MATERIALS TABLE





## Abbreviations

ABC: Advanced breast cancer
AE: Adverse event
AUC_0-24_: Area under the curve from zero to 24 hours
AUC_inf_: Area under the curve from time zero to infinity
BLRM: Bayesian logistic regression model
C_max_: Maximal drug concentration
CTCAE: Common Terminology Criteria for AEs
DDS: Dose-determining set
DLT: Dose limiting toxicities
ECOG: Eastern Cooperative Oncology Group
EWOC: Escalation with overdose control
FAS: Full analysis set
GI: Gastrointestinal
HER2–: Human epidermal growth factor receptor 2-negative
HNSCC: Head and neck squamous cell carcinoma
HR+: Hormone receptor-positive
MTD: Maximum tolerated dose
mTOR: Mammalian target of rapamycin
PIK3CA: Phosphatidylinositol-4,5-bisphosphate 3-kinase catalytic subunit alpha
PK: Pharmacokinetic
QD: Once daily
QW: Once weekly
RECIST: Response Evaluation Criteria In Solid Tumors
RP2D: Recommended Phase II dose
T_max_: Time to maximum concentration
