# Supplementary material for: A Phase Ib, open-label, dose-finding study of alpelisib in combination with paclitaxel in patients with advanced solid tumors
Source: Oncotarget. 2018 Aug 3;9(60):31709–18. doi: 10.18632/oncotarget.25854 (PMC6114962; doi:10.18632/oncotarget.25854)
Supplement: Supplementary file 1 [file oncotarget-09-31709-s001.pdf]

A Phase Ib, open-label, dose-finding study of alpelisib in combination with paclitaxel in patients with advanced solid tumors

SUPPLEMENTARY MATERIALS

Supplementary Table 1: Criteria for defining dose-limiting toxicities[a].

See Supplementary File 1
