# Supplementary material for: A Phase Ib, open-label, dose-finding study of alpelisib in combination with paclitaxel in patients with advanced solid tumors
Source: Oncotarget. 2018 Aug 3;9(60):31709–18. doi: 10.18632/oncotarget.25854 (PMC6114962; doi:10.18632/oncotarget.25854)
Supplement: Supplementary file 2 [file oncotarget-09-31709-s002.docx]

**Supplementary Table 1:** Criteria for defining dose-limiting toxicities^a^.

| **Toxicity** | **DLT Criteria** |
| --- | --- |
| Blood and lymphatic system disorders | Febrile neutropenia CTCAE Grade ≥3 |
| Cardiac disorders | Cardiac toxicity CTCAE Grade ≥3 or cardiac event that is symptomatic or requires medical intervention |
|  | Clinical signs of cardiac disease, such as unstable angina or myocardial infarction, or Troponin CTCAE Grade 3 (confirmed with a repeat Troponin within 24 hrs) |
|  | ECG QTc interval prolonged CTCAE Grade ≥3 |
| Vascular disorders: Hypertension | Persistent hypertension CTCAE Grade ≥3 requiring more than one drug or more intensive therapy than previously administered |
| General disorders and administration site conditions | Fatigue CTCAE Grade ≥3 for >7 consecutive days |
| Skin and subcutaneous tissue disorders^b^: Rash and/or photosensitivity | Rash or photosensitivity CTCAE Grade 3 for >7 consecutive days despite skin toxicity treatment |
|  | Rash or photosensitivity CTCAE Grade 4 |
| Metabolism and nutrition disorders: Hyperglycemia | Hyperglycemia Grade 2 (FPG >160–250 mg/dL; 8.89–13.89 mmol/L) (confirmed with a repeat FPG within 24 hrs) that does not resolve to grade ≤1 (<140 mg/dL; <7.8 mmol/L) within 21 consecutive days (after initiation of oral antidiabetic treatment) |
|  | Hyperglycemia Grade 3 (FPG >250 mg/dL; >13.89 mmol/L) (confirmed with a repeat FPG within 24 hrs) for >7 consecutive days despite oral antidiabetic treatment |
|  | Hyperglycemia Grade 4 (FPG >500 mg/dL; >27.8 mmol/L) |
|  | Hyperglycemia leading to diabetic keto-acidosis, hospitalization for IV insulin infusion, or non-ketotic coma |
| Neuropathy/pain | ≥CTCAE grade 3 peripheral sensory or motor neuropathy |
| GI disorders^b^ | Diarrhea CTCAE Grade ≥3 ≥48 hrs, despite the use of antidiarrhea therapy |
|  | Nausea/vomiting CTCAE Grade ≥3 ≥48 hrs, despite the use of antiemetic therapy |
|  | Pancreatitis CTCAE Grade ≥3 |
| Investigations^c^ | Total blood bilirubin CTCAE Grade 2 for >7 consecutive days |
|  | Total blood bilirubin CTCAE Grade ≥3 |
|  | AST or ALT CTCAE Grade ≥3 in conjunction with total blood bilirubin CTCAE Grade ≥ 2 of any duration |
|  | AST or ALT CTCAE Grade ≥3 for >7 consecutive days  AST or ALT CTCAE Grade 4 |
|  | Serum alkaline phosphatase CTCAE Grade 4 for >7 consecutive days |
|  | Serum lipase and/or serum amylase (asymptomatic) CTCAE Grade 3 for >7 consecutive days |
|  | Serum lipase and/or serum amylase (asymptomatic) CTCAE Grade 4 |
|  | Serum creatinine CTCAE Grade ≥3 |
|  | Neutrophil count CTCAE Grade ≥3 for >7 consecutive days |
|  | Platelet count CTCAE Grade 3 for >7 consecutive days and/or with signs of bleeding |
|  | Platelet count CTCAE Grade 4 |
|  | Hypomagnesaemia CTCAE Grade 3 for >3 consecutive days and not correctable with supplements, or symptomatic |
|  | Hypomagnesaemia CTCAE Grade 4 |
| Other hematologic & nonhematologic toxicities | Any other CTCAE Grade ≥3 toxicity except:  Lymphocyte count decreased (lymphopenia) CTCAE Grade ≥3 unless clinically significant |

AE, adverse event; ALT, alanine aminotransferase; AST, aspartate aminotransferase;
CTCAE, Common Terminology Criteria for Adverse Event version 4.03; DLT, dose-limiting toxicity; ECG, Electrocardiogram; FPG, fasting plasma glucose; hrs, hours; IV, intravenous;
QTc, Q-T interval in the ECG.

^a^ Apart from the criteria listed above, if a lower grade AE led to a dose interruption of more than 7 consecutive days of alpelisib, or two or more consecutive doses of paclitaxel within Cycle 1, or between Cycle 1 and Cycle 2 Day 1, this AE was considered a DLT. Hypersensitivity reactions were not considered DLTs. However, patients experiencing severe hypersensitivity reaction during Cycle 1 despite adequate premedication would be discontinued from the study. ^b^ Patients did not initially receive prophylactic treatment for skin toxicity or nausea/vomiting during Cycle 1. However, prophylactic treatment may have been initiated in all patients at the dose level if these toxicities were observed and in all further patients if at least one patient had experienced skin toxicity or nausea/vomiting CTCAE Grade ≥3 or if at least two patients experienced skin toxicity or nausea/vomiting CTCAE Grade ≥2. However anti-emetics may have been applied for treatment if the patient experienced nausea/vomiting CTCAE Grade ≥1, at the discretion of the physician. ^c^ For any CTCAE Grade 3 or 4 hepatic toxicity that did not resolve within 7 days to CTCAE Grade ≤1 (or CTCAE Grade ≤2 if liver infiltration with tumor present), an abdominal CT scan must have been performed to assess if it was related to disease progression.
